# Supplementary material for: The causal relationship between the human gut microbiota and pyogenic arthritis: a Mendelian randomization study
Source: Front Cell Infect Microbiol. 2024 Nov 26;14:1452480. doi: 10.3389/fcimb.2024.1452480 (PMC11629706; doi:10.3389/fcimb.2024.1452480)
Supplement: Supplementary file 1 [file DataSheet1.zip › mendelian test/ebi-a-GCST90027625.csv_forest.pdf]

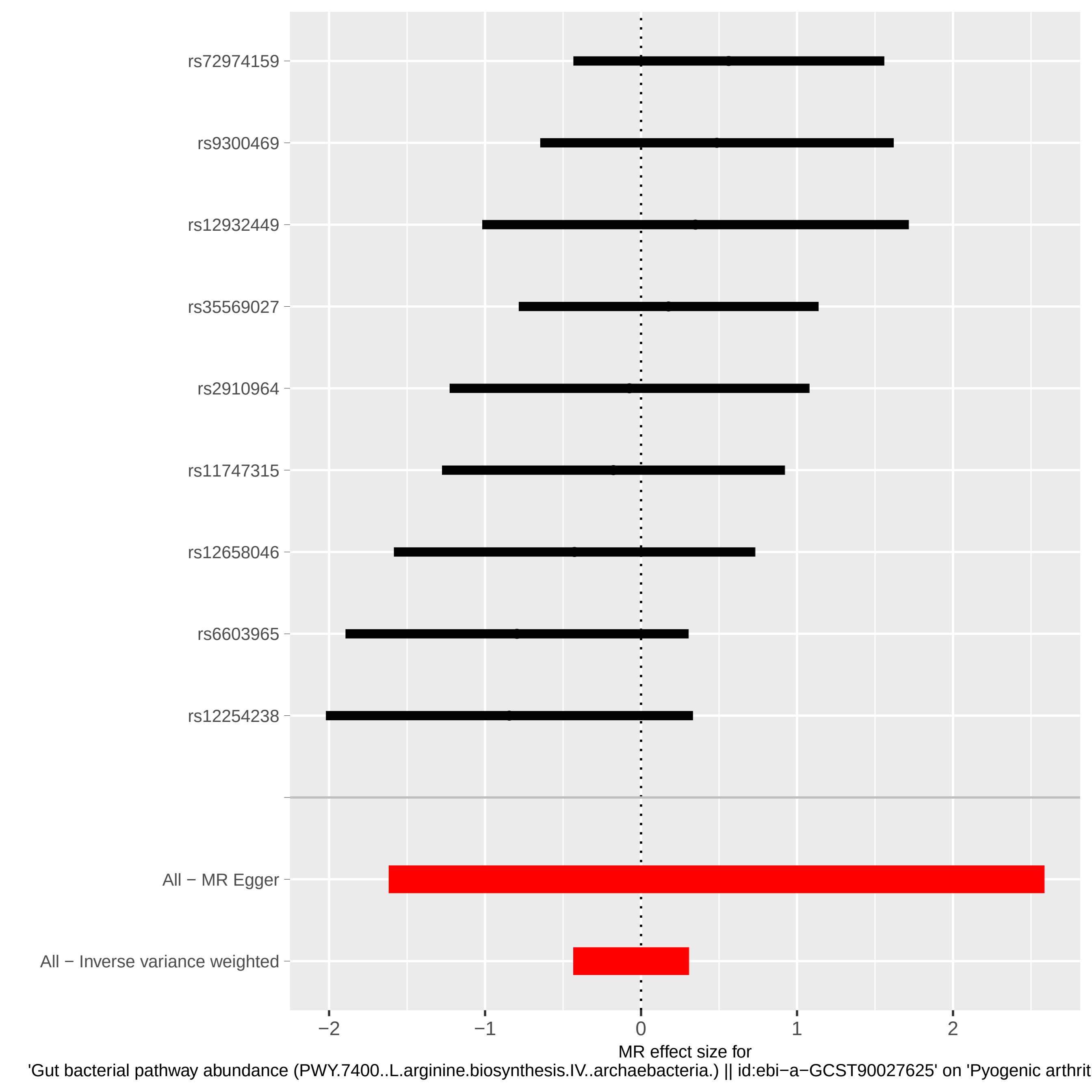
rs72974159
rs9300469
rs12932449
rs35569027
rs2910964
rs11747315
rs12658046
rs6603965
rs12254238
All − MR Egger
All − Inverse variance weighted
−2
−1
0
1
2
MR effect size for
'Gut bacterial pathway abundance (PWY.7400..L.arginine.biosynthesis.IV..archaebacteria.) || id:ebi−a−GCST90027625' on 'Pyogenic arthrit
